# Drug poisonings following bariatric surgery: Case series report

**DOI:** 10.1002/bcp.70387

**Published:** 2025-12-15

**Authors:** Eman Mshari, Fannie LaJeunesse‐Trempe, Stephen Morley, Caroline Copeland

**Affiliations:** ^1^ Centre for Pharmaceutical Medicine Research, Institute of Pharmaceutical Science King's College London London UK; ^2^ Faculty of Pharmacy Laval University Quebec Canada; ^3^ Chemical Pathology and Toxicology Unit University Hospital Leicester Leicestershire UK; ^4^ National Programme on Substance Use Mortality London UK

**Keywords:** bariatric surgery, drug absorption, drug‐related death, gastrointestinal tract, toxicity

## Abstract

Bariatric surgery alters gastrointestinal anatomy and physiology, which likely impacts upon oral medication absorption. Drug‐ and alcohol‐related deaths in this population are being increasingly reported; however, toxicological detail is lacking. Using data reported to the National Programme on Substance Use Mortality, we identified 18 deaths in people who had previously undergone bariatric surgery. Opioids were detected in almost all cases and were frequently implicated in causing death. Multiple medications were detected at post‐mortem in every case and often included medications that the deceased was not actively prescribed. Mental health conditions and chronic pain were commonly listed comorbidities and one‐third of deaths were deemed suicide. Illicit drug and alcohol use was rare, highlighting a distinct vulnerability to prescription medication harms. Our findings emphasize a need to understand how bariatric surgery alters pharmacokinetics and for integrated, multidisciplinary aftercare, therapeutic drug monitoring and tailored education of safe medication use in bariatric surgery patients.

**What is already known about this subject**

- Bariatric surgery alters gastrointestinal physiology which likely impacts oral medication absorption.
- Bariatric surgery patients often have high incidence of mental health and chronic pain conditions, leading to long‐term antidepressant and analgesic use.
- Emerging evidence shows increased risk of drug‐ and alcohol‐related harms, including overdose and suicide, often several years post‐surgery.

**What this study adds**

- The first toxicology‐based case series of overdose deaths in bariatric surgery patients, showing opioids as the main contributors.
- Identifies that illicit drug and alcohol use was rare, highlighting a distinct vulnerability to prescription medication harms.
- Demonstrates need for multidisciplinary aftercare and education of safe medication use in bariatric surgery patients.

## 1 INTRODUCTION

Bariatric surgery is a well‐established treatment for obesity.^1^, ^2^, ^3^, ^4^, ^5^ There are several different bariatric surgery procedures, with gastric bypass and sleeve gastrectomy the most commonly performed.^4^ By design these procedures reduce food intake and absorption to achieve weight loss. However, their substantial alteration of the gastrointestinal anatomy and concomitant physiology will markedly affect the absorption of oral medications.^6^ This may lead to reduced plasma concentrations^7^ or alternatively supratherapeutic concentrations that carry risk of toxicity.^6^, ^8^

Published evidence indicates that drug‐ and alcohol‐related harms and deaths are increasing in bariatric surgery patients, with most deaths occurring several years postoperatively and as a result of unintentional overdose, although intentional overdose has also been documented.^9^, ^10^, ^11^, ^12^, ^13^, ^14^, ^15^ Prescription opioids represent a consistent pathway to harm, with chronic pain reported by significant proportions of patients both preoperatively and postoperatively^16^, ^17^, ^18^; this risk is likely compounded by a high prevalence of mental health issues in this patient cohort.^19^, ^20^ However, most evidence has been derived from administrative datasets that lack toxicological detail, with limited ability to distinguish between prescribed *vs*. non‐prescribed medications or illicit substance use.

The National Programme on Substance Use Mortality (NPSUM) collects coroner reports on deaths involving psychoactive substances across England, Wales and Northern Ireland. Using this dataset, we examined deaths in post‐bariatric surgery patients, describing their demographics, clinical profiles, substances detected and prescribing patterns. This may provide insight into overdose risk and inform targeted prevention strategies in this patient population.

## 2 METHODS

### 2.1 NPSUM

Established in 1997, the NPSUM collates death reports related to psychoactive drug use from coroners in England, Wales and Northern Ireland. Coroners voluntarily report to NPSUM if psychoactive drugs were detected and/or implicated in causing the death or if the decedent had a history of drug use.

Coroners collate information from a variety of sources, including statements from witnesses, family and friends; reports from first responders; post‐mortem and toxicology reports; and healthcare records from general practitioners, hospitals and drug and alcohol services.

### 2.2 Study design

We retrospectively identified relevant NPSUM cases reported between 1997 and 1 March 2025 where decedents had a past medical history of bariatric surgery by searching the cause(s) of death, accident details and past medical/social history fields for the following terms: ‘balloon’, ‘band’, ‘bariatric’, ‘biliopancr*’, ‘bypass’, ‘gastr*’, ‘roux’, ‘sleeve’ and ‘weight’. We screened the structured fields to exclude cases that did not meet inclusion criteria.

### 2.3 Data analysis

Data collection, analysis and statistics were conducted using IBM® SPSS™ version 29 and Microsoft Excel 365. Summary statistics from all cases reported to the NPSUM were also retrieved for comparative interpretations.

### 2.4 Ethics

The King's College London Biomedical & Health Sciences, Dentistry, Medicine and Natural & Mathematical Sciences Research Ethics Sub‐Committee reconfirmed (August 2025) that analyses of NPSUM data do not require ethics review as all subjects are deceased.

## 3 RESULTS

Eighteen decedents with a bariatric surgery history were identified. Gastric bypass was the most common procedure that had been performed (67% of cases, *n* = 12/18), followed by gastric band placement (*n* = 2/18) and sleeve gastrectomy (*n* = 1). In three cases, the procedure performed was not specified.

### 3.1 Post‐mortem, implicated and prescribed drugs

Multiple substances were detected at post‐mortem in all cases (median 6; range 4–11). All cases featured licensed medications, with only two cases featuring illicit substances (cannabis *n* = 2/18, cocaine *n* = 1/18). Alcohol was detected in four cases but in all instances at a level below the drink‐drive limit in England (mean 42.5 ± 18.2 mg/dL, range 19–63 mg/dL; note: alcohol detections attributed to post‐mortem production [≤10 mg/dL in blood]^21^ were excluded). Opioids were the most commonly detected drug class featuring in all but one case (94.4% of cases, *n* = 17/18), with antidepressants the second most commonly detected (83.3% of cases, *n* = 15/18), followed by non‐opioid analgesics (72.2% of cases, *n* = 13/18; Table 1).

**TABLE 1:** Drugs detected at post‐mortem, implicated in causing death and psychoactive medications prescribed to decedents.

	Post‐mortem					Total implicated					Implication by source					
	*n*	%	Cases with quantifications	Concentration (mg/L)		*n*	%	Cases with quantifications	Concentration (mg/L)		Prescribed		Not prescribed		Source unknown	
				Median	Range				Median	Range	*n*	%	*n*	%	*n*	%
Total cases	18	100				18	100				17	100	17	100	1	100
Unlicensed substances																
Alcohol	4	22.22	4	440	190–630	‐	‐	‐	‐	‐	‐	‐	‐	‐	‐	‐
Illicit substances	2	11.1	‐	‐	‐	‐	‐	‐	‐	‐	‐	‐	‐	‐	‐	‐
Cannabis	2	11.1	1	0.0009	0.0009	‐	‐	‐	‐	‐	‐	‐	‐	‐	‐	‐
Cocaine	1	5.6	1	6.60	6.60	1	5.6	1	6.6	6.6	‐	‐	1	5.9	‐	‐
Licensed psychoactive medications																
Antidepressants	15	83.3	‐	‐	‐	8	44.4	‐	‐	‐	6	35.3	1	5.9	1	100
Amitriptyline	7	38.9	5	0.21	0.02–2.39	4	22.2	2	2.13	1.87–2.39	2	11.8	1	5.9	1	100
Duloxetine	1	5.6	1	1.10	1.10	1	5.6	1	1.10	1.10	1	5.9	‐	‐	‐	‐
Fluoxetine	1	5.6	1	0.03	0.03	‐	‐	‐	‐	‐	‐	‐	‐	‐	‐	‐
Mirtazapine	2	11.1	1	1.20	1.20	2	11.1	1	1.20	1.20	2	11.8	‐	‐	‐	‐
Sertraline	3	16.7	3	1.10	0.41–1.40	‐	‐	‐	‐	‐	‐	‐	‐	‐	‐	‐
Venlafaxine	5	27.8	5	1.79	0.03–15.54	4	22.2	4	2.00	0.03–15.54	3	17.6	‐	‐	1	100
Anti‐psychotics	6	33.3	‐	‐	‐	5	27.8	‐	‐	‐	4	23.5	‐	‐	1	100
Chlorpromazine	1	5.6	1	5.20	5.20	1	5.6	1	5.20	5.20	1	5.9	‐	‐	‐	‐
Quetiapine	5	27.8	4	0.93	0.43–1.90	4	22.2	3	1.04	0.83–1.90	3	17.6	‐	‐	1	100
Zuclopenthixol	1	5.6	‐	‐	‐	‐	‐	‐	‐	‐	‐	‐	‐	‐	‐	‐
Benzodiazepines and Z‐drugs	9	50.0	‐	‐	‐	5^c^	27.8	‐	‐	‐	4	23.5	2	11.8	‐	‐
Alprazolam	1	5.6	1	0.14	0.14	1	5.6	1	0.14	0.14	‐	‐	1	5.9	‐	‐
Diazepam	6	33.3	3	0.11	0.07–0.46	1	5.6	‐	‐	‐	‐	‐	1	5.9	‐	‐
Zolpidem	1	5.6	1	0.29	0.29	1	5.6	1	0.29	0.29	1	5.9	‐	‐	‐	‐
Zopiclone	5	27.8	5	0.23	0.02–0.49	3	16.7	3	0.38	0.23–0.49	3	17.6	‐	‐	‐	‐
Gabapentinoids	10	55.6	‐	‐	‐	7	38.9	‐	‐	‐	3	17.6	3	17.6	1	100
Gabapentin	3	16.7	3	76.00	22.00–89.00	3	16.7	3	76.00	22.00–89.00	1	5.9	2	11.8	‐	‐
Pregabalin	7	38.9	5	14.30	5.40–100.50	4	22.2	2	57.40	14.30–100.50	2	11.8	1	5.9	1	100
Opioids	17	94.4	‐	‐	‐	17^c^	94.4	‐	‐	‐	8	47.1	9	52.9	1	100
Codeine	9	50.0	8	1.70	0.01–7.76	7	38.9	7	2.20	0.13–7.76	5	29.4	2	11.8	‐	‐
Dextropropoxyphene	1	5.6	1	0.30	0.30	‐	‐	‐	‐	‐	‐	‐	‐	‐	‐	‐
Dihydrocodeine	3	16.7	2	5.53	3.56–7.50	2	11.1	2	5.53	3.56–7.50	‐	‐	2	11.8	‐	‐
Dipipanone	1	5.6	‐	‐	‐	‐	‐	‐	‐	‐	‐	‐	‐	‐	‐	‐
Fentanyl	1	5.6	1	0.0063	0.0063	1	5.6	1	0.0063	0.0063	1	5.9	‐	‐	‐	‐
Hydrocodone	1	5.6	‐	‐	‐	‐	‐	‐	‐	‐	‐	‐	‐	‐	‐	‐
Morphine	5	27.8	4	0.19	0.05–0.36	7^b^	38.9	6	0.14	0.02–0.36	5	29.4	2	11.8	‐	‐
Oxycodone	3	16.7	3	0.56	0.08–0.58	3	16.7	3	0.56	0.08–0.58	‐	‐	2	11.8	1	100
Tramadol	5	27.8	5	6.50	0.51–9.71	4	22.2	4	7.25	4.28–9.71	1	5.9	3	17.6	‐	‐
Sedating antihistamine	4	22.2	‐	‐	‐	‐	‐	‐	‐	‐	‐	‐	‐	‐	‐	‐
Cyclizine	4	22.2	3	0.30	0.29–2.59	‐	‐	‐	‐	‐	‐	‐	‐	‐	‐	‐
Diphenhydramine	1	5.6	‐	‐	‐	‐	‐	‐	‐	‐	‐	‐	‐	‐	‐	‐
Licensed non‐psychoactive medications																
Anti‐cholinergics	1	5.6	‐	‐	‐	1	5.6	‐	‐	‐	1	5.9	‐	‐	‐	‐
Procyclidine	1	5.6	‐	‐	‐	1	5.6	‐	‐	‐	1	5.9	‐	‐	‐	‐
Cardiovascular medications	5	27.8	‐	‐	‐	2	11.1	‐	‐	‐	2	11.8	‐	‐	‐	‐
Propranolol	4	22.2	2	4.7	4.30–5.00	2	11.1	2	4.65	4.30–5.00	2	11.8	‐	‐	‐	‐
Bisoprolol	1	5.6	‐	‐	‐	‐	‐	‐	‐	‐	‐	‐	‐	‐	‐	‐
Non‐opioid analgesics	13	72.2	‐	‐	‐	1	1.0	‐	‐	‐	1	5.9	‐	‐	‐	‐
Aspirin	1	5.6	‐	‐	‐	‐	‐	‐	‐	‐	‐	‐	‐	‐	‐	‐
Diclofenac	1	5.6	‐	‐	‐	‐	‐	‐	‐	‐	‐	‐	‐	‐	‐	‐
Ibuprofen	2	11.1	‐	‐	‐	‐	‐	‐	‐	‐	‐	‐	‐	‐	‐	‐
Naproxen	3	16.7	‐	‐	‐	‐	‐	‐	‐	‐	‐	‐	‐	‐	‐	‐
Paracetamol	10	55.6	3	37.5	31.00–217.00	1	5.6	1.0	31.00	31.00	1^a^	5.9	‐	‐	‐	‐
Other	7	38.9	‐	‐	‐	‐	‐	‐	‐	‐	‐	‐	‐	‐	‐	‐
Cetirizine	1	5.6	‐	‐	‐	‐	‐	‐	‐	‐	‐	‐	‐	‐	‐	‐
Dexamethasone	1	5.6	‐	‐	‐	‐	‐	‐	‐	‐	‐	‐	‐	‐	‐	‐
Lansoprazole	1	5.6	‐	‐	‐	‐	‐	‐	‐	‐	‐	‐	‐	‐	‐	‐
Lignocaine	1	5.6	‐	‐	‐	‐	‐	‐	‐	‐	‐	‐	‐	‐	‐	‐
Metoclopramide	1	5.6	‐	‐	‐	‐	‐	‐	‐	‐	‐	‐	‐	‐	‐	‐
Omeprazole	1	5.6	‐	‐	‐	‐	‐	‐	‐	‐	‐	‐	‐	‐	‐	‐
Piperacillin	1	5.6	‐	‐	‐	‐	‐	‐	‐	‐	‐	‐	‐	‐	‐	‐
Quinine	1	5.6	‐	‐	‐	‐	‐	‐	‐	‐	‐	‐	‐	‐	‐	‐
Sildenafil	1	5.6	‐	‐	‐	‐	‐	‐	‐	‐	‐	‐	‐	‐	‐	‐

^a^ Implicated as part of a co‐codamol formulation.

^b^ In two cases where morphine was implicated, its presence was attributed to its formation as an active metabolite of codeine.

^c^ In one case two drugs within the same class were implicated, one which was prescribed, one which was not prescribed.

Similarly, opioids were the drug class most commonly implicated in causing death (94.4% of cases, *n* = 17/18), followed by antidepressants (44.4% of cases, *n* = 8/18; Table 1). In only one case was a non‐opioid analgesic implicated in causing death, and in this instance, it was implicated as the combined preparation co‐codamol. The third most commonly implicated drug class was the gabapentinoids (38.9% of cases, *n* = 7/18; Table 1). In the single case where cocaine was detected, it was implicated in causing death, with a post‐mortem blood concentration of 6.6 mg/L, which is within the fatal range.^22^, ^23^ Both THC detections were considered incidental post‐mortem findings.

Where prescribing history was known (94.4% of cases, *n* = 17/18), psychoactive medication was prescribed in 16 cases (median 3.5 medications; range 1–7). One or more of these medications was implicated in causing death in 81.3% of these cases (*n* = 13/16). Medications without an active prescription were present in most cases (70.6%, *n* = 12/17).

### 3.2 Underlying cause and manner of death

All deaths were deemed to have been caused by acute drug toxicity. Regarding manner of death, 12 were determined to have been accidental in nature (75% of cases), five as suicides (27.8% of cases) and one of undetermined intent (5.6% of cases).

### 3.3 Past medical history

A past medical history was provided in all cases. There was a high prevalence of mental health conditions (77.8% of cases, *n* = 14/18; Table 2) with depression the most commonly cited (33.3% of cases, *n* = 6/18). There was also high incidence of chronic pain (50.0% of cases, *n* = 9/18), with 22.2% citing a history of previous overdose (*n* = 4/18). Obstructive sleep apnoea was listed as a comorbidity in two cases (11.1%) and chronic obstructive pulmonary disease (COPD) in three cases (16.7%).

**TABLE 2:** Summary characteristics of past medical histories of bariatric surgery patients.

	*n*	%
History of mental health	14	77.8
Depression	6	33.3
Anxiety	3	16.7
Unspecified condition	4	22.2
Suicidal ideation	2	11.1
Self‐harm	1	5.6
Low self‐esteem	1	5.6
PTSD	1	5.6
Chronic pain	9	50.0
Previous overdose	4	22.2

### 3.4 Demographics

There were more deaths in females at complete contrast to the all NPSUM trend (*X* ^2^ *p* < 0.001; Table 3), with bariatric case decedents significantly older (Student's *t* test *p* < 0.05; Table 3). Where information was provided (83.3% of cases, *n* = 15/18), bariatric case decedents were majority living with other people and unemployed (Table 3). Where indices of multiple deprivation could be obtained (94.4% of cases, *n* = 17/18), bariatric case decedents were majority living in the most deprived areas (Table 3). In only one bariatric case was there a known history of drug use, a significantly lower rate than the all NPSUM cohort (*X* ^2^ *p* < 0.001; Table 3).

**TABLE 3:** Summary characteristics of demographics of bariatric surgery patients.

	Bariatric case series			All NPSUM
	*n*	%	Valid %	Valid %
Total	18	100	100	100
Age (years; mean ± SD [range])	47.2 ± 9.1 (31–60)			40.9 ± 13.4 (0–105)
Sex				
Male	5	27.8	154.3	73.2
Female	13	72.2	401.2	26.8
Employment status				
Unemployed	7	38.9	46.7	54.4
Employed	4	22.2	26.7	30.5
Retired/invalidity/sickness	3	16.7	20.0	11.4
Childcare/houseperson	1	5.6	6.7	1.6
Student/pupil	‐	‐	‐	2.1
Not known	3	16.7	‐	‐
Living circumstances				
With others	10	55.6	66.7	41.3
Alone	5	27.8	33.3	46.4
No fixed abode/hostel	‐	‐	‐	7.1
Other	‐	‐	‐	5.2
Not known	3	16.7	‐	‐
Deprivation quintile^a^				
1	7	41.2	41.2	47.6
2	4	23.5	23.5	21.3
3	2	11.8	11.8	14.9
4	2	11.8	11.8	9.4
5	2	11.8	11.8	6.8
Drug use history				
Yes (IVDU)	1 (‐)	5.6 (‐)	10.0 (‐)	72.9 (17.2)
No	9	50.0	90.0	27.1
Not known	8	44.4	‐	‐

^a^ One person living in Jersey for which a deprivation quintile was not available.

## 4 DISCUSSION

We have presented a case series of post‐bariatric surgery patients who died due to drug overdose. The high incidence of opioid involvement, together with frequent detection of other analgesics, suggests that this patient population faces specific risks regarding pharmacological pain management. When combined with a high incidence of antidepressant use, mental health problems and suicide in a predominantly female cohort, this reflects a vulnerable population requiring integrated aftercare to mitigate risks of both intentional and unintentional overdose.

### 4.1 Altered pharmacokinetics

Most opioids are weak bases (pKa ~ 7.5–9^24^). In the acidic fasted stomach (pH ~ 1–3),^25^ they are largely ionized which limits their absorption.^26^ Bariatric surgery alters this environment, as resection or bypass of stomach acid‐producing regions reduces hydrochloric acid output.^27^ In addition, stomach pH can be further increased upon alkaline bile reflux from the small intestine into the gastric pouch.^28^, ^29^ Indeed, post‐bariatric surgery fasting gastric pH values have been reported in the pH ~ 4–6 range.^30^ The reduced acidity of gastric contents entering the duodenum can therefore increase proximal small intestinal pH.^31^, ^32^ Collectively, these changes may facilitate the absorption of weak base opioids in both the stomach and small intestine, enhancing their toxicity profile. Given that most antidepressants possess similar physicochemical properties,^33^ comparable effects leading to their increased absorption may also occur.

However, bariatric surgery also accelerates gastric emptying^34^, ^35^, ^36^ and reduces small intestine surface area,^36^, ^37^ shortening the oral drug absorption window. Extended‐release formulations (e.g., OxyContin®, MS Contin® and Effexor XR®) may therefore deliver subtherapeutic exposure.^38^ Conversely, higher gastric pH may trigger premature dissolution of extended‐release preparations that utilize pH‐dependent polymer coatings designed to dissolve only once exposed to pHs in the ~5.5–6.5 range^38^ potentially triggering premature or erratic drug release; such ‘dose dumping’^39^ may cause transient supratherapeutic plasma concentrations, with associated risks of toxicity.

Taken together, these physiological and anatomical alterations mean that bariatric surgery may unpredictably increase or decrease systemic drug exposure, depending on the surgical procedure, the drug's physicochemical properties and its formulation, which may be contributing to the high rate of accidental overdose observed in this cohort. While quantifications were performed for some of the drugs detected, the small sample size, unknown drug formulations and doses and variable intervals between death and sampling unfortunately preclude firm conclusions regarding altered pharmacokinetics or differences from concentrations reported in non‐surgical overdose cases.

### 4.2 High mental health and chronic pain burden

Depression, anxiety and antidepressant use were common in this cohort, consistent with previous literature reporting high psychiatric morbidity pre‐surgery and post‐surgery.^19^, ^20^ While bariatric procedures often improve obesity‐related comorbidities (e.g., type 2 diabetes and heart disease),^1^, ^2^, ^3^, ^4^, ^5^ psychological outcomes are more variable, with some patients experiencing persistent or worsening mood symptoms: a recent meta‐analyses showed increased long‐term suicide risk compared with the general population.^19^ In this study, one‐third of deaths met Office for National Statistics suicide criteria (all deaths deemed suicide and of undetermined intent in those aged ≥10^40^), which alongside frequent histories of previous overdose indicates a population with increased mental health vulnerability. Other obesity‐related comorbidities, namely, obstructive sleep apnoea and COPD, were also present in a minority of cases and were not considered contributory to death.

Half of the cases reported chronic pain, aligning with evidence that musculoskeletal pain, neuropathic pain and pain syndromes often persist post‐weight loss.^16^, ^17^, ^18^ This often results in prolonged analgesic use,^16^, ^41^ which matches the drugs most commonly detected at post‐mortem in this study. The combination of mental health diagnoses and chronic pain can foster polypharmacy,^41^ and—as described above—bariatric surgery can alter drug pharmacokinetics such that standard doses may produce higher peak plasma concentrations,^6^, ^42^ increasing toxicity risk, or alternatively, lower peak concentrations,^6^, ^42^ prompting repeated dosing that can elevate risk of adverse effects. With regard to antidepressants, subtherapeutic dosing may fail to achieve adequate symptom control and elevate suicide risk.

### 4.3 Prescription drug vulnerability in a low illicit drug use cohort

In this case series, illicit drug use was rare, alcohol use was low and there was only one case with a documented history of drug use. Despite this, prescription medications—often without active prescriptions—were frequently implicated in causing death. This suggests that, while not engaging in typical patterns of dependent or recreational drug use,^43^, ^44^, ^45^ these patients' medicalized background, and chronic pain and mental health conditions may have facilitated their access to and confidence in using prescription medications, thus creating a distinct risk profile for self‐directed prescription drug use. Conventional harm reduction initiatives, which majority target illicit drug use,^46^ will therefore likely not be successful if applied to this population. Given the routine long‐term clinical follow‐up inherent to post‐bariatric surgical care,^47^ there is opportunity to integrate long‐term monitoring and preventive interventions into appointments, with proactive management of mental health, chronic pain and medication use providing a practical and targeted approach to educate patients on drug safety.

### 4.4 Future research

Pharmacokinetic changes post‐bariatric surgery are poorly understood, and current evidence is insufficient for prescribing guidance.^6^ Until standardized studies are performed to evaluate the impact of different bariatric surgery techniques, a clinical pharmacologist should be involved in the bariatric surgery multidisciplinary meetings where prospective patients are discussed, and therapeutic drug monitoring should be routine for patients post‐surgery.^42^

### 4.5 Limitations

The NPSUM relies on voluntary coroner reports and incomplete past medical histories, so bariatric patient deaths are likely underestimated.

## 5 CONCLUSION

Bariatric surgery patients face unique overdose risks due to altered pharmacokinetics and comorbidities of chronic pain and mental health conditions. Prescribers should be cautious when initiating or adjusting opioid, antidepressant or other pH‐/formulation‐sensitive medications. Despite low illicit drug and alcohol use, prescription medications were commonly implicated in causing death, reflecting a vulnerability linked to medicalized knowledge and ongoing healthcare requirements. Conventional harm reduction initiatives for drug use are unlikely to be effective in this patient population; instead, integrated aftercare addressing mental health, pain and medication safety, with tailored patient education, may be more effective in reducing harm.

## AUTHOR CONTRIBUTIONS

**Eman Mshari:** Validation; formal analysis; investigation; writing—original draft; writing—review and editing; visualization. **Fannie LaJeunesse‐Trempe:** Conceptualization; writing—review and editing. **Stephen Morley:** Conceptualization; writing—review and editing. **Caroline Copeland:** Conceptualization; methodology; validation; resources; data curation; writing—review and editing; supervision; project administration; funding acquisition.

## CONFLICT OF INTEREST STATEMENT

The authors have no conflicts of interest to declare.

## Data Availability

The data that support the findings of this study are available upon reasonable request from the principal investigator. The data are not publicly available due to privacy restrictions.
